# Dementias Platform UK: Bringing genetics into life

**DOI:** 10.1002/alz.13782

**Published:** 2024-03-20

**Authors:** Ganna Leonenko, Sarah Bauermeister, Dipanwita Ghanti, Joshua Stevenson‐Hoare, Emily Simmonds, Keeley Brookes, Kevin Morgan, Nishi Chaturvedi, Paul Elliott, Alan Thomas, Nicholas Wareham, John Gallacher, Valentina Escott‐Price

**Affiliations:** ^1^ Dementia Research Institute Cardiff University Cardiff UK; ^2^ Dementias Platform UK Department of Psychiatry University of Oxford Oxford UK; ^3^ Division of Psychological Medicine and Clinical Neurosciences School of Medicine Cardiff University Cardiff UK; ^4^ Biosciences School of Science and Technology Nottingham Trent University Nottingham UK; ^5^ University Nottingham Nottingham UK; ^6^ MRC Unit for Lifelong Health & Ageing, UCL London UK; ^7^ MRC Centre for Environment and Health School of Public Health Imperial College London London UK; ^8^ UK Dementia Research Institute Imperial College London London UK; ^9^ Translational and Clinical Research Institute Newcastle University Newcastle UK; ^10^ MRC Epidemiology Unit University of Cambridge Cambridge UK

**Keywords:** dementia, genetic data, harmonization, imputation, polygenic risk scores

## Abstract

**INTRODUCTION:**

The Dementias Platform UK (DPUK) Data Portal is a data repository bringing together a wide range of cohorts. Neurodegenerative dementias are a group of diseases with highly heterogeneous pathology and an overlapping genetic component that is poorly understood. The DPUK collection of independent cohorts can facilitate research in neurodegeneration by combining their genetic and phenotypic data.

**METHODS:**

For genetic data processing, pipelines were generated to perform quality control analysis, genetic imputation, and polygenic risk score (PRS) derivation with six genome‐wide association studies of neurodegenerative diseases. Pipelines were applied to five cohorts.

**DISCUSSION:**

The data processing pipelines, research‐ready imputed genetic data, and PRS scores are now available on the DPUK platform and can be accessed upon request though the DPUK application process. Harmonizing genome‐wide data for multiple datasets increases scientific opportunity and allows the wider research community to access and process data at scale and pace.

## 1 BACKGROUND

Dementias Platform UK (DPUK; https://www.dementiasplatform.uk/) brings together a wide range of cohorts in the DPUK Data Portal to facilitate collaborative research opportunities and answer important questions about dementia.^1^ DPUK is fully auditable with a remote access platform that contains > 60 population and clinical cohorts across a range of imaging, genetic, and survey (e.g., physical, psychosocial, and cognitive) data. The aggregation of individual datasets in such a platform maximizes their utility and enables joint analyses of complex data, which increases power and provides a shared and secure environment without the risk of disclosing sensitive information.

Individual genetic data are not easy to share between studies due to the EU's General Data Protection Regulation (GDPR), in which genetic data are included in the list of sensitive data. Only secure computational platforms (like DPUK) with a legally compliant (ISO 27001) process of data handling and processing offer an opportunity to combine the genetic data from a number of studies.

Access to individual levels of genetic data provides a new independent resource not only to explore neurodegenerative diseases such as different types of dementias, Parkinson's disease (PD), and amyotrophic lateral sclerosis (ALS) from different research angles, but also perform joint analyses with the aim to uncover additional genetic associations and/or insights into relevant biological mechanisms. Recent advances in genome wide association studies (GWAS) have made an enormous contribution and provide valuable insights about the pathogenesis of neurodegenerative disease, providing a positive step forward for the development of disease‐modifying treatments.^2^ A polygenic risk score (PRS) approach that combines small additive effects of specific loci across the genome has become an increasingly powerful tool to help identify individuals at higher/lower risk of developing complex disorders. Furthermore, a PRS approach could also help explain the proportion of genetic variance that seems to be missing when focusing only on genome‐wide significant hits. It has shown great potential in Alzheimer's disease (AD) prediction with accuracy^3^, ^4^ can be used for studying genetic overlap among disorders of the brain.^5^, ^6^

The DPUK platform is a unique collection of studies which were historically collected in the UK over the past 50 years to answer specific research questions. The studies are complementary to other large UK cohorts (UK Biobank,^7^ Genomics England^8^). With a rapidly increasing number of GWAS studies, there is a lack of independent studies that can be used for replication, polygenic risk scoring, and other analyses requiring sample independence. Until recently, the DPUK platform has been a large, valuable, but underused resource. The lack of homogeneity of the phenotypic and genotypic information makes it difficult to use and therefore data harmonization is crucial to leverage its full potential.

In this paper, we set an example of combining genetic data across five studies that were approved for this project and provide research‐ready datasets to the wider community that can be compared and/or analyzed together. This has been achieved by the creation and installation of standardized processing pipelines on the DPUK Portal including quality control (QC) steps, genetic imputation, and calculation of standardized PRSs with the six latest GWAS summary statistics related to neurodegeneration diseases, namely AD,^9^ AD‐by‐proxy, PD,^10^ frontotemporal dementia,^11^ ALS,^12^ and Lewy body dementia.^13^ All generated and QC‐ed data are provided in a widely accepted PLINK format.^14^ The pipelines are set as a series of commands in a bash script and can be easily modified if any additional data filtering is required. To perform other genetic analyses, software packages can be requested to be installed by the DPUK technical support team. Detailed information about the data application process to access DPUK cohorts and processed data is available on the DPUK portal (https://portal.dementiasplatform.uk/Apply). The associated phenotypic data processing and harmonization is ongoing. The ready‐to‐use, harmonized, and QC‐ed data offers an advantage to researchers to accelerate collaborative projects, remove the need to repeatedly curate the data on per‐project basis, and reduce the cost of data management and the level of uncertainty in the choice of analytical methodology. All data, pipelines, and PRS scores can be accessed and used within the DPUK platform by other researchers.

**RESEARCH IN CONTEXT**

1. **Systematic review**: The authors have undertaken a comprehensive review of the literature using traditional (e.g., PubMed) sources. The relevant references were added to the paper describing the DPUK portal, cohorts, and data analysis methodology.
2. **Interpretation**: We generated and installed pipelines within the DPUK portal for quality‐control, genetic imputation, and polygenic risk score (PRS) calculation. Pipelines, imputed genetic data, and PRS will be available for investigators via the DPUK platform, where individual study data access consent and pre‐approved ethics permit such data sharing (upon data owner approval).
3. **Future directions**: Given the important value of data sharing from both a scientific and funder's perspective, it would be inappropriate for the scientific community not to continue offering and using these valuable resources, while ensuring compliance with the permissions and ethics of individual studies. This work allows the wider research community to access and process data at scale and pace.

## 2 METHODS

### 2.1 Access data on DPUK

**FIGURE 1:**
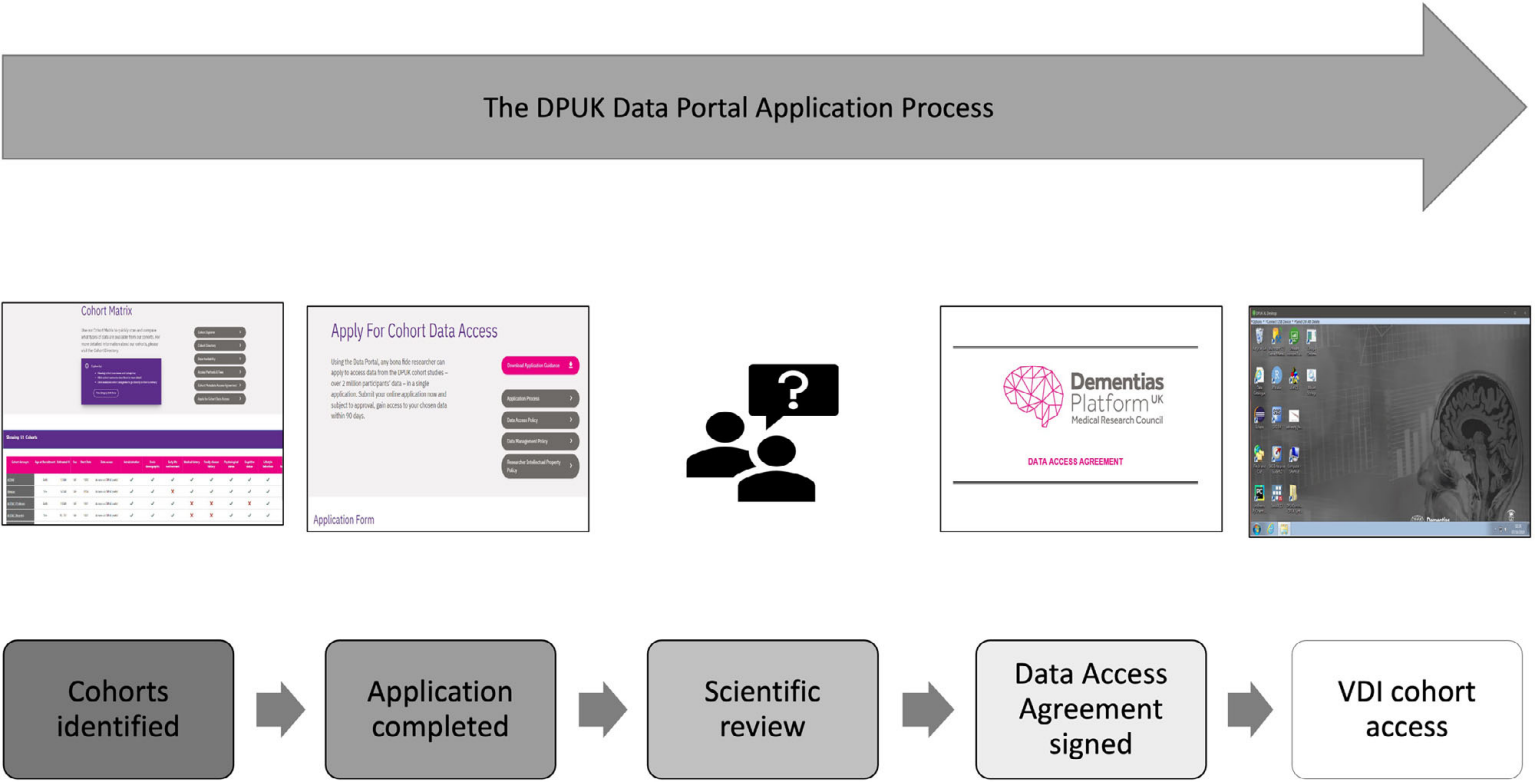
Flowchart of DPUK application process. DPUK, Dementias Platform UK; VDI, virtual desktop interface.

Bona fide academic and industry researchers are allowed to apply for access to the DPUK cohort datasets. Upon approval of an application and signing of a Data Access Agreement (https://portal.dementiasplatform.uk/Apply), researchers access approved datasets on a virtual desktop interface (VDI) within the DPUK Data Portal. All statistical packages and tools are preinstalled in the VDI and data cannot be downloaded. Figures, summary statistics, and graphs may be downloaded for publication and presentation purposes. Scripts may be uploaded onto the VDI. The flowchart of DPUK application process can be seen in Figure 1.

### 2.2 Studies with genetics

For this project we used DPUK cohorts that agreed to participate in sharing the individual‐level genotype data with the main aim of merging and processing the datasets together. These cohorts were also used to test the data processing pipelines and provide research‐ready datasets for analyses by the individual cohorts, thereby encouraging collaboration among the studies. All cohorts had basic demographic information (sex, age, ethnicity), and most of the cohorts had cognitive tests and neurodegenerative disease diagnoses (clinical or *post mortem*). The cognitive assessments, however, were measured using different questionnaires, depending on the purpose of the study. The work to harmonize and standardize the phenotypic cognitive information is ongoing.

Ethical approval was not required as this was obtained at source by the cohort and only secondary analysis was undertaken.

Brains for Dementia Research (BDR)^15^, ^16^ is an initiative that has recruited participants across five UK brain banks to help to investigate the mechanistic pathways of dementia by studying phenotypic data collected during their lives and their donated brain tissue after death. BDR data collection is ongoing with > 3200 people signed up to donate their brains. We used the BDR data freeze as of October 2020, including participants aged 56 to 104. The data collection has followed standardized operating procedures of brain donations along with standard longitudinal clinical and psychometric assessments and genetic data.

Generation Scotland (GS) of the Scottish Family Health Study is a family‐based genetic epidemiology study with DNA and socio‐demographic and clinical data from > 20,000 volunteers across Scotland aged 18 to 98 years, from February 2006 to March 2011.^17^ Participants and their families were invited to take part in the study with the aim to investigate links between genetics and common complex familial diseases such as cardiovascular disease, cognitive decline, mental illnesses, and so forth.

Epic Norfolk (EN) is a part of the European Prospective Investigation into Cancer (EPIC), a large multi‐center cohort study with participants enrolled from 23 centers across Europe, EN being one of them. More than 30,000 people living in Norwich and surrounding towns and rural areas were recruited into the EN study between 1993 and 1997 who were aged between 39 and 79.^18^ The data include dietary and lifestyle information, health questionnaires, numerous disease diagnoses, and genetics.

The Medical Research Council National Survey of Health and Development (NSHD) is the longest‐running British birth cohort (1946) from England, Scotland, and Wales. Five thousand three hundred sixty‐two participants were recruited at birth in a single week in March 1946,^19^ with > 2800 people in the active sample. Information that has been collected includes lifestyle, environmental, childhood health and development, lifetime social circumstances, genetic, and imaging data.

The Airwave Health Monitoring Study (AW) is a longitudinal epidemiological study of the police force to evaluate possible health risks associated with use of TETRA, a digital communication system used by police forces and other emergency services in Great Britain since 2001,^19^, ^20^ with 42,112 participants recruited by the end of 2012. The cohort has been richly phenotyped and has blood and urine samples, lifestyle factors, health screening, mental health, and well‐being measurements and genetics. Summary of available genetics for these cohorts can be seen in Table 1.

**TABLE 1:** Genetic description of cohorts.

Cohort abbreviation	Cohort full name	Genetic data received	*N* SNPs	*N* samples	Phenotypes
BDR	Brains for Dementia Research	Neurochip	478,633	570	Sociodemographic, cognitive status, mental health, MMSE
GS	Generation Scotland	GS_SFHS, CHR(X), *APOE*	604,858 17,574,2	20,032 20,110	Sociodemographic, mental health, cognitive test, cognitive status
EN	EPIC Norfolk	Axiom 2020	728,244	21,041	
NSHD	Medica Research Council National Survey of Health and Development	NeuroX2 Imputed with HRC	11,081,207	2864	Amyloid status, brain measurements, MMSE
AW1	The Airwave Health Monitoring Study	Affymetrix	845,487	4493	Sociodemographic, cognitive status, mental health
AW2		IlluminacoreExome	542,677	14,887	

Abbreviations: *APOE*, apolipoprotein E; MMSE, Mini‐Mental State Examination; SNP, single nucleotide polymorphism.

### 2.3 Genetic data harmonization

Before any joint genetic analysis, the data should be merged on overlapping single nucleotide polymorphisms (SNPs), harmonized, and checked for outliers. Originally, there were a total of 32,365 overlapping SNPs among five datasets (BDR, GS, EN, AW1, AW2) that were genotyped on different platforms (see Table S1 in supporting information). This significantly limits the capacity to conduct any genome‐wide study at a SNP, gene, or haplotype level or construct PRSs across all studies.

We developed and installed a genotype QC and imputation pipeline to facilitate standardized procedures for all aspects of genetic data and it is now available on the DPUK platform. We have chosen a standard protocol^21^ for QC analysis with widely used PLINK^14^ and R software. The choice of thresholds for each QC step was not too stringent to retain the majority of individuals and genetic variants. However, (1) these thresholds can be adjusted within the pipeline if more stringent/relaxed inclusion criteria are required; (2) additional filtering steps can be applied by researchers on already QC‐ed cohorts; and (3) additional software can be requested to be installed and applied to perform other genetic analyses, for example, to re‐calculate kinship scoring.

The pipeline is initiated with pre‐imputation QC checks that were applied to the all‐target cohorts. Samples were removed based on call rate <95%; heterozygosity (HET > ± 0.1); relatedness based on identity by descent with PI_HAT > 0.2, except the GS cohort. We did not exclude related individuals in the GS sample, as the family members were specifically recruited according to the study design. All cohorts were merged with the 1000 Genomes dataset to conduct a principal component analysis (PCA). Individuals were removed if they did not cluster near the 1000 Genomes European cluster. SNPs were removed with minor allele frequency (MAF) < 0.01; Hardy–Weinberg equilibrium (HWE) *P*_HWE_ ≤ 10^−6^; with missing data proportion >5%. At the pre‐imputation step, SNPs were aligned with the 1000 Genomes reference panel, hg19. SNP alignment included removing SNPs that have discordant information present with the reference panel (i.e., allele mismatch, strand flips, etc.). The pre‐imputation QC steps and exclusions for each cohort are presented in Tables S2–S7 in supporting information and PCA are presented in Figure S1A–F in supporting information.

**FIGURE 2:**
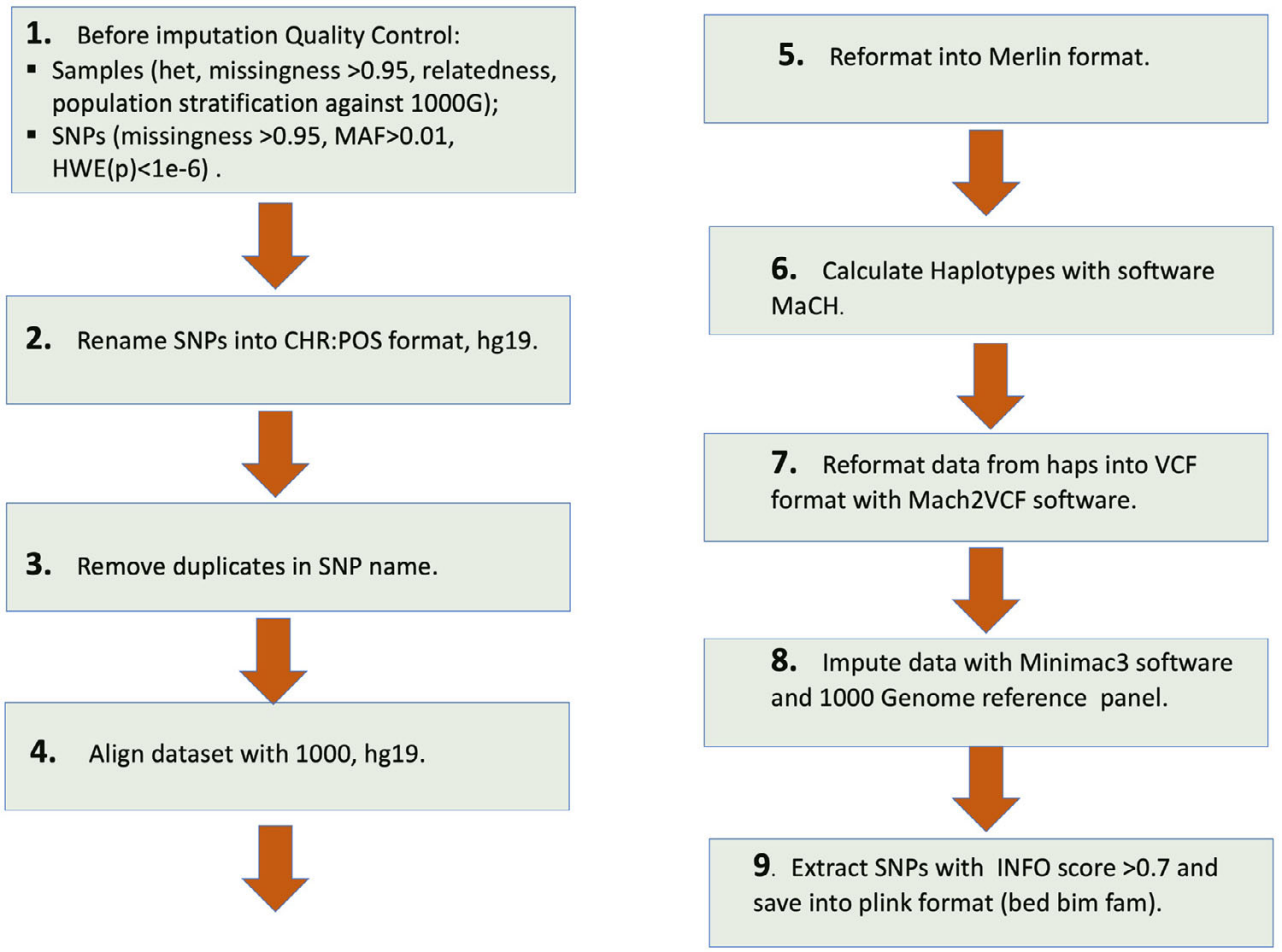
Workflow of the imputation protocol for genotyped data. HWE, Hardy–Weinberg equilibrium; MAF, minor allele frequency; SNP, single nucleotide polymorphism.

In the next step, the Minimac imputation tool^22^, ^23^ was implemented. This tool relies on a two‐step approach: (1) phasing samples into a series of estimated haplotypes with MaCH software^24^ and (2) using the derived haplotypes for genotype imputation. The 1000 Genomes reference panel (https://www.internationalgenome.org) in VCF format was used because it is publicly available for download onto the DPUK platform. We did not use HRC^25^ or TOPMED^26^ reference panels due to limitations induced by the data‐sharing policy. The detailed workflow of the imputation protocol is represented in Figure 2.

The last step of the pipeline, post‐imputation QC, was applied to remove variants with imputation information scores < 0.7, MAF < 0.01, and *P*_HWE_ ≤ 10^−6^.

### 2.4 Derivation of PRSs

PRS derivation requires discovery GWAS summary statistics (effect sizes, reference alleles, and *P* values) and target data, which is independent of the GWAS with individual level genetic information available for each sample.

Before proceeding with PRS calculations, we uploaded to the DPUK Portal publicly available GWAS summary statistics for the six largest neurodegenerative disease studies: (1) clinical AD GWAS of 63,926 samples^9^ (AD); (2) AD‐by‐proxy/clinical GWAS and related dementias (ADRD) of 487,511 samples;^27^ (3) Parkinson's Disease GWAS (PD) of 1,474,097 samples;^10^ (4) Frontotemporal Dementia GWAS (FTD) of 12,928 samples;^11^ (5) Amyotrophic Lateral Sclerosis GWAS (ALS) of 138,086 samples;^12^ and (6) Lewy Body Dementia GWAS (LBD) of 6618 samples.^13^ In each set of GWAS summary statistics, we reformatted the variant IDs into “rs numbers,” aligned them to the 1000 Genomes reference panel, and removed variants with standard error (SE) > 2 in the corresponding summary statistics. PRS was calculated for both all available SNPs and for all SNPs excluding *APOE* region (chromosome 19:44.4‐46.5Mb) using AD and ADRD summary statistics (PRS.no.APOE).

Because there is still a debate about the comparability of various PRS approaches and optimal *P* value threshold, we have chosen the PRS approach with continuous shrinkage (PRS‐CS)^28^ that does not depend on *P* value threshold or clumping parameters and shows improved predictive accuracy across a wide range of disorders with complex genetic structure.^29^ PRS‐CS retains more SNPs and reduces information loss, compared to the widely used linkage disequilibrium (LD) clumping methods that only retain one lead SNP in an LD block.^30^, ^31^

**FIGURE 3:**
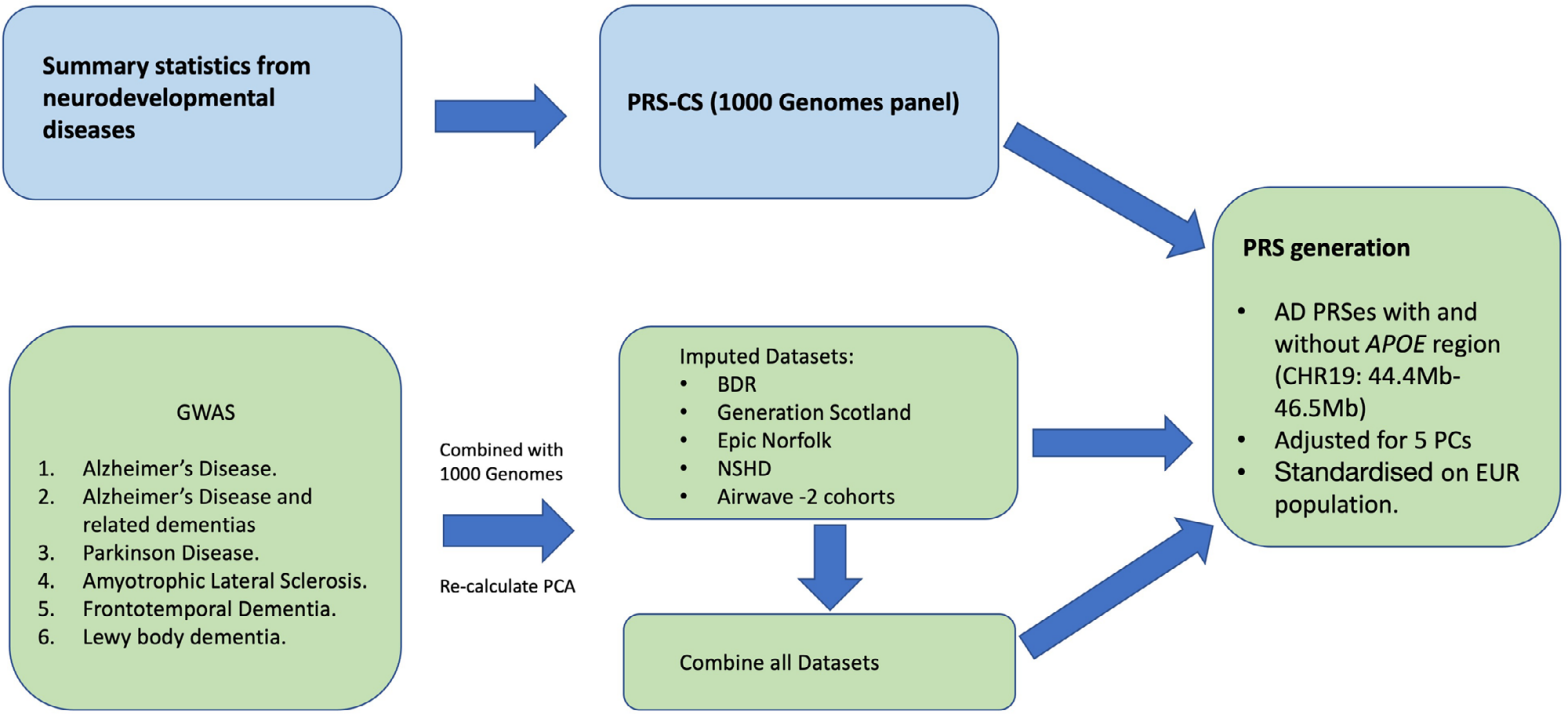
Workflow of the PRS generation protocol. *APOE*, apolipoprotein E; BDR, Brains for Dementia Research cohort; NSHD‐MRC Medical Research Council National Survey of Health and Development cohort; PCA, principal component analysis; PC, principal components; PRS‐CS, polygenic risk score approach calculated with 1000 Genomes reference panel.

In the pipeline, PRS‐CS scores were generated with six GWAS summary statistics for each cohort separately and on the combined dataset. The derived scores were adjusted for five principal components (PCs). We adopted the approach of PRS standardization, which allows scores to be comparable between studies.^31^ For that, each cohort was merged with 1000 Genomes European population (*N* = 503) and we standardized the cohorts’ PRS using the mean and standard deviation (SD) of the PRS from 1000 Genomes European population. The PRS calculation diagram can be seen in Figure 3. To investigate the difference between PRS distributions, the Kolmogorov–Smirnov test was applied, and *P* value was considered significant after Bonferroni correction for multiple testing (*P* ≤1.4e‐3 = 0.05/36).

## 3 RESULTS

### 3.1 Imputation

An overview of pre‐imputation QC results, imputation, and post‐imputation QC results that were performed for each cohort and final number of samples and variants are represented in Tables S2–S8 in supporting information. The six DPUK cohorts were imputed and QC‐ed and are ready to be disseminated with pre‐computed 5 PCs (with and without 1000 Genomes European population). The combination of six cohorts provides us with a dataset of 60,522 individuals on 4,037,483 variants, common among the cohorts.

### 3.2 PRS for each study

Imputed and QC‐ed genetic data was used for PRS score calculations and the scores are ready to be disseminated to other research projects. PRS‐CS scores were generated for each cohort (BDR, GS, EN, NSHD, AW1, AW2), adjusted for PCs and standardized against 1000 Genomes European population, as described in Section 2. It can be observed that all PRS, as expected, have an approximately normal distribution; and cohorts’ and European 1000 Genomes’ PRS distributions are closely matched; see Figure S2A–F in supporting information.

### 3.3 PRS distributions in combined study

**FIGURE 4:**
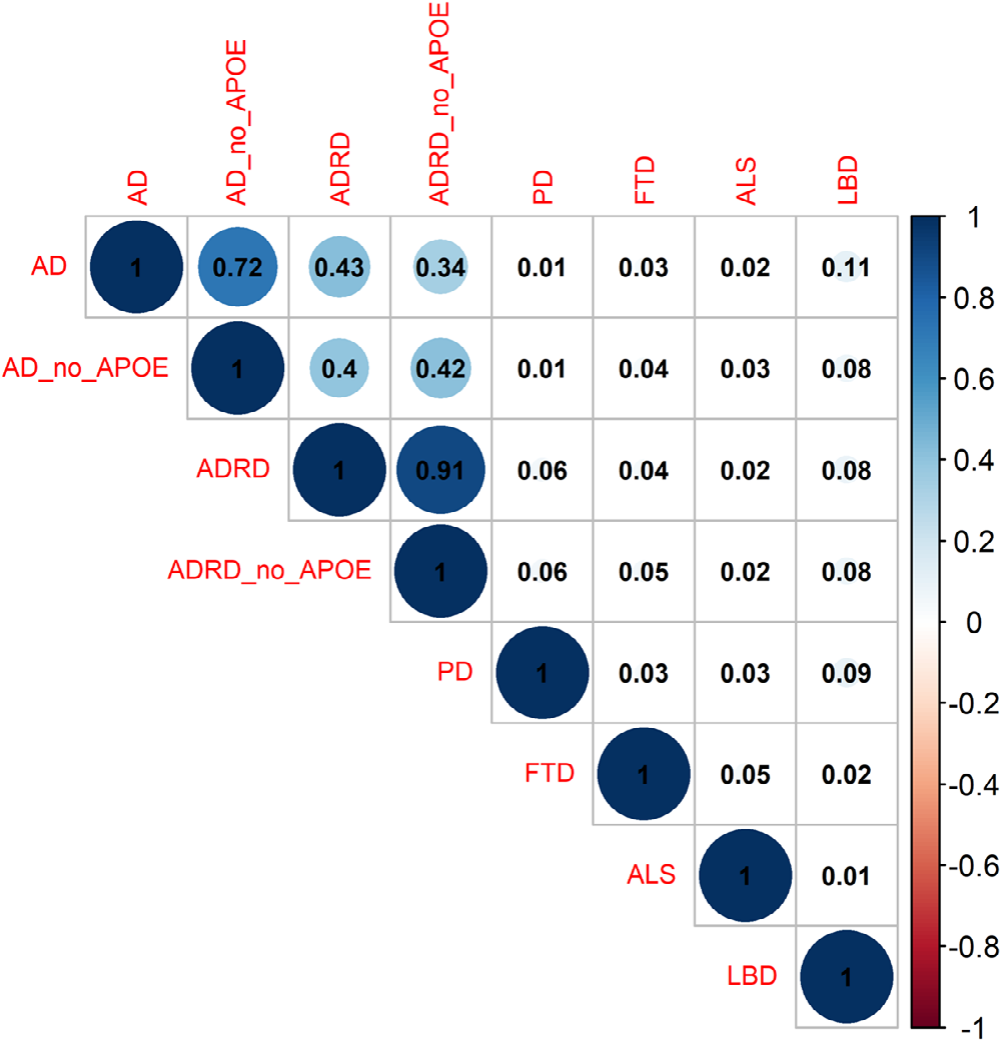
Matrix of Pearson's correlation of PRS‐CS scores that have been calculated with six GWAS summary statistics: AD, ADRD, FTD, PD, ALS, LBD, with and without I region (AD, ADRD) in the combined cohort. *APOE*, apolipoprotein E; AD—clinical Alzheimer's disease GWAS; AD_no_APOE, Alzheimer's disease GWAS without *APOE* region; ADRD—Alzheimer's disease clinical/proxy GWAS and related dementias; ADRD_no_APOE—Alzheimer's disease clinical/proxy GWAS without *APOE* region; ALS, amyotrophic lateral sclerosis GWAS; FTD, frontotemporal dementia GWAS; GWAS, genome‐wide association study; LBD, Lewy body dementia; PD, Parkinson's disease GWAS.

First, we examined Pearson's correlations among all PRS‐CS scores that were calculated for six neurodegenerative diseases. Figure 4 shows that the highest correlations (*r* between 0.34 and 0.91) can be observed between PRS calculated with AD and ADRD GWAS and depend on the inclusion of the *APOE* region. Correlation between AD and LBD PRS reached *r* = 0.11, while with other GWAS (PD, ALS) *r* is < ± 0.1. Note, that LBD‐PRS correlates the most with both AD/ADRD and PD‐PRS (0.11 and 0.09, respectively) and is in line with LBD diagnosis,^13^ in which people with LBD have problems with understanding, thinking, memory, and judgement, similar to AD.

**FIGURE 5:**
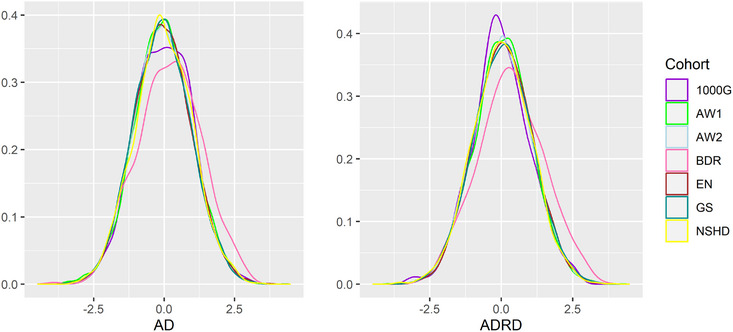
Standardized PRS distributions calculated with AD (left) and ADRD (right) summary statistics on combined dataset split by cohort (BDR, EN, GS, NSHD, AW1, AW2, 1000G). 1000G, 1000 Genomes European population cohort; AD, Alzheimer's disease; ADRD, Alzheimer's disease and related dementias; AW, Airwave Health Monitoring Study BDR, Brains for Dementia Research; EN, Epic Norfolk; GS, Generation Scotland; NSHD, Medical Research Council National Survey of Health and Development.

Next, we investigated PRS distributions of the combined dataset generated with six neurodegenerative GWAS (AD, ADRD, PD, ALS, FTD, LBD); see Figure S3A–F in supporting information with the corresponding Kolmogorov–Smirnov test *P* values in Table S9 in supporting information. Figure 5 presents standardized PRS distributions calculated with AD and ADRD summary statistics for each DPUK cohort. All PRS have similar to 1000 Genomes (purple line) normal distribution, with the exception of the BDR study (pink line) that is shifted to the right in both cases. Indeed, BDR is a case–control study (with pathologically confirmed diagnosis) and is enriched with dementia cases compared to other cohorts, which are population based. The difference between PRS distributions (BDR and 1000 Genomes) is border‐line significant (*P* = 6.5 × 10^−3^) with AD‐PRS and significant (*P* = 1.1 × 10^−5^) with ADRD‐PRS; see Table S9.

## 4 DISCUSSION

The DPUK Data Portal has been designed to aggregate data from research groups across the United Kingdom and internationally into a single platform to maximize their utility and enable joint analysis of complex data that can lead to advancing new discoveries. Sharing genetic data is particularly challenging due to its identifiability, which requires protection and confidentiality but is of the utmost importance while requiring compliance with the permissions and ethics of each individual cohort. Given the complexity and heterogeneity of the genetic data due to genotyping platforms, differences in QC analyses, and the number of overlapping variants, when combined at the individual level, joint analysis is only possible after standardization and imputation of the data.

We have established a series of pipelines that involve (1) QC analysis prior to imputation, (2) imputation with the 1000 Genomes reference panel, (3) post‐imputation QC analysis, and (4) calculation of PRS with the six latest and largest GWAS summary statistics of neurodegenerative disorders.

The data processing pipelines were installed with standard QC and data analysis parameters and are open‐source scripts which can be easily adjusted by other researchers, suitable for the needs of their study designs. The pipelines can also be modified to perform other genetic analyses, that is, gene‐set/pathway‐specific PRS calculation with other GWAS summary statistics.

Our study has some limitations. First, for the PRS derivation, the independence between GWAS and the target dataset is required as even small sample overlap can produce significantly inflated results.^32^ We were unable to analytically assess the sample overlap between GWAS and the DPUK datasets as only GWAS summary statistics are publicly available. However, to our best knowledge, there is no overlap between DPUK cohorts and the GWAS studies we have used.

Second, despite boosted statistical power, ADRD GWAS generated with clinically assessed AD cases that were meta‐analyzed with “AD‐by‐proxy” approach^27^ (AD diagnosis is based on participants’ self‐reported diagnosis for their parents) may have limitations that include imprecision of diagnosis, heterogeneity in the survey, and systematic biases related to UK Biobank sample collection.^33^, ^34^, ^35^

Third, the resulting number of SNPs shared between all DPUK cohorts is limited (≈ 4 M), compared to other imputed datasets. This number is reduced because the NSHD study used NeuroX2 array for genotyping (with a small number of overlapping SNPs with any of the imputation reference panels). However, we provide imputed genetic data for each cohort separately on the DPUK Portal, which is equivalent to the expected number of imputed SNPs (8,9 million).

Finally, for the imputation, we have used the 1000 Genomes (publicly available) reference panel, as the DPUK data sharing policy does not allow any data to leave the platform, whereas the imputation with the TOPMED panel was only possible when the data moves to the Imputation Server provided by the University of Michigan (USA). We, however, used the same software and similar pipeline as implemented at the Michigan server.

In summary, imputed genetic data, the combined dataset, and PRS are now available for investigators via the DPUK Data Portal, where the individual study data access consent and pre‐approved ethics permit such data sharing upon approved application. Given the important value of data sharing from both a scientific and funder's perspective, we encourage researchers to use these data as it would be inappropriate for the scientific community not to continue offering and using these valuable resources.

## CONFLICT OF INTEREST STATEMENT

All authors have declared no conflicts of interest. Author disclosures are available in the supporting information.

## CONSENT STATEMENT

All human subjects provided consent for participation with the source cohort. This consent included data collection and repurposing for secondary data analysis. Full ethical approvals had been obtained at source by the originating cohort according to their ethical approval body. For this study, additional ethical approval was not required as only secondary analysis was undertaken on anonymized secondary data from pre‐consented human subjects.

## Supporting information

Supporting Information

Supporting Information
